# 21-(3-Carboxy­propano­yl)-11β,17-dihydroxy­pregn-4-ene-3,20-dione monohydrate

**DOI:** 10.1107/S160053681001648X

**Published:** 2010-05-12

**Authors:** Hongliang Wang, Junhai Xiao, Pengfei Chen, Tie-Min Sun

**Affiliations:** a School of Pharmaceutical Engineering, Shenyang Pharmaceutical University, Shenyang 110016, People’s Republic of China; b Beijing Institute of Pharmacology and Toxicology, Beijing 100850, People’s Republic of China

## Abstract

In the title compound, C_25_H_34_O_8_·H_2_O, the two crylohexane rings adopt chair conformations. In the crystal, the organic mol­ecule and the water mol­ecule are linked by O—H⋯O hydrogen bonds, generating a three-dimensional network.

## Related literature

For background to glucocorticoids, see: Schäcke *et al.* (2002 ▶). For the synthesis, see: Fang *et al.* (2007 ▶).
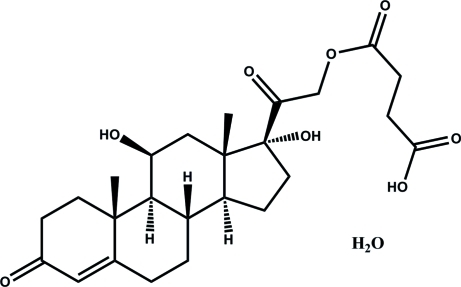


## Experimental

### 

#### Crystal data

C_25_H_34_O_8_·H_2_O
*M* *_r_* = 480.54
Orthorhombic, 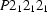

*a* = 7.2672 (15) Å
*b* = 16.606 (3) Å
*c* = 20.009 (4) Å
*V* = 2414.7 (8) Å^3^
*Z* = 4
Mo *K*α radiation
μ = 0.10 mm^−1^
*T* = 293 K
0.16 × 0.14 × 0.13 mm

#### Data collection

Rigaku R-AXIS RAPID IP diffractometer
21231 measured reflections
2848 independent reflections
2562 reflections with *I* > 2σ(*I*)
*R*_int_ = 0.051

#### Refinement

*R*[*F* ^2^ > 2σ(*F* ^2^)] = 0.039
*wR*(*F* ^2^) = 0.106
*S* = 1.06
2848 reflections
309 parameters
H-atom parameters constrained
Δρ_max_ = 0.16 e Å^−3^
Δρ_min_ = −0.17 e Å^−3^

### 

Data collection: *CrystalClear* (Rigaku/MSC, 2005 ▶); cell refinement: *CrystalClear*; data reduction: *CrystalClear*; program(s) used to solve structure: *SHELXS97* (Sheldrick, 2008 ▶); program(s) used to refine structure: *SHELXL97* (Sheldrick, 2008 ▶); molecular graphics: *SHELXTL* (Sheldrick, 2008 ▶); software used to prepare material for publication: *SHELXTL*.

## Supplementary Material

Crystal structure: contains datablocks I, global. DOI: 10.1107/S160053681001648X/hb5414sup1.cif

Structure factors: contains datablocks I. DOI: 10.1107/S160053681001648X/hb5414Isup2.hkl

Additional supplementary materials:  crystallographic information; 3D view; checkCIF report

## Figures and Tables

**Table 1:** Hydrogen-bond geometry (Å, °)

*D*—H⋯*A*	*D*—H	H⋯*A*	*D*⋯*A*	*D*—H⋯*A*
O2—H2⋯O8^i^	0.82	2.06	2.828 (3)	155
O3—H3*A*⋯O9	0.82	1.91	2.720 (2)	170
O7—H7⋯O1^ii^	0.82	1.91	2.716 (2)	167
O9—H2*W*⋯O2^iii^	0.85	2.08	2.884 (3)	158
O9—H1*W*⋯O1^iv^	0.85	1.95	2.795 (3)	174

Symmetry codes: (i) 
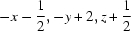
; (ii) 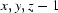; (iii) 
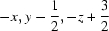
; (iv) 
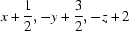
.

**Fig. 1.**
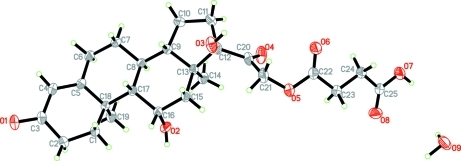
Crystal structure of (I) with displacement ellipsoids shown at the 50% probability level.

## supplementary crystallographic information

### Comment

Glucocorticoids (GCs) process varied biological properties such as anti-inflammatory, immunosuppressive and countershock activities (Schäcke *et al.*, 2002). In view of these importances and to determine the molecular conformation, a crystallographic study of the title compound has been carried out.

The molecular structure is shown in Fig. 1. All the bond lengths and angles are within normal ranges. The molecule contains three six-membered rings (A ring atoms C1-C5/C18; B ring atoms C5-C8/C17/C18; C ring atoms C8/C9/C13/C15-C17) and a five-member ring (D ring atoms C9-C13). Ring B and C adopt chair conformations.

In the crystal structure, the molecules are linked *via* intermolecular O2—H2···O8, O7—H7···O1 interactions. The molecules and water are additionally linked by strong O3—H3A···O9, O9—H2W···O2, O9—H1W···O1 actions (Fig. 2).

### Experimental

To a solution of hydrocortisone (5.43 g, 15 mmol) in pyridine (75 ml), succinic anhydride (3 g, 30 mmol) was added dropwise with stirring at room temperature. The mixture was refluxed for 8 hrs. After pouring the solution to ice water (100 ml) and adjusting pH to 5.0-5.5 with 5% HCl (aq), the resulting white solid was obtained and then collected by filtration, washed with water and dried (Fang *et al.*, 2007). Colourless prisms of (I) were recrystalized from ethanol by the slow evaporation of the solvent at room temperature after several days, presumably the water of crystallisation was absorbed from the atmosphere.

### Refinement

Anomalous dispersion was negliglble and Friedel pairs were merged before refinement. H atoms of water molecule were located in a difference Fourier map and then refined in riding mode with *U*_iso_(H)=1.5*U*_eq_(O).All the other H atoms were placed in idealized locations as riding atoms, with *U*_iso_(H) = 1.2*U*_eq_(C) for methylene, *U*_iso_(H) = 1.5*U*_eq_(C) for methyl groups and *U*_iso_(H) = 1.5*U*_eq_(O) for hydroxyl groups.

### Crystal data

**Table:**

C_25_H_34_O_8_·H_2_O	*F*(000) = 1032
*M**_r_* = 480.54	*D*_x_ = 1.322 Mg m^−^^3^
Orthorhombic, *P*2_1_2_1_2_1_	Mo *K*α radiation, λ = 0.71073 Å
Hall symbol: P 2ac 2ab	Cell parameters from 17248 reflections
*a* = 7.2672 (15) Å	θ = 3.1–27.5°
*b* = 16.606 (3) Å	µ = 0.10 mm^−^^1^
*c* = 20.009 (4) Å	*T* = 293 K
*V* = 2414.7 (8) Å^3^	Prism, colourless
*Z* = 4	0.16 × 0.14 × 0.13 mm

### Data collection

**Table:**

Rigaku R-AXIS RAPID IP diffractometer	2562 reflections with *I* > 2σ(*I*)
Radiation source: rotating anode	*R*_int_ = 0.051
graphite	θ_max_ = 26.5°, θ_min_ = 3.1°
oscillation scans	*h* = −8→9
21231 measured reflections	*k* = −20→20
2848 independent reflections	*l* = −25→25

### Refinement

**Table:**

Refinement on *F*^2^	Primary atom site location: structure-invariant direct methods
Least-squares matrix: full	Secondary atom site location: difference Fourier map
*R*[*F*^2^ > 2σ(*F*^2^)] = 0.039	Hydrogen site location: inferred from neighbouring sites
*wR*(*F*^2^) = 0.106	H-atom parameters constrained
*S* = 1.06	*w* = 1/[σ^2^(*F*_o_^2^) + (0.063*P*)^2^ + 0.2837*P*] where *P* = (*F*_o_^2^ + 2*F*_c_^2^)/3
2848 reflections	(Δ/σ)_max_ < 0.001
309 parameters	Δρ_max_ = 0.16 e Å^−^^3^
0 restraints	Δρ_min_ = −0.17 e Å^−^^3^

### Special details

**Table:**

Geometry. All esds (except the esd in the dihedral angle between two l.s. planes) are estimated using the full covariance matrix. The cell esds are taken into account individually in the estimation of esds in distances, angles and torsion angles; correlations between esds in cell parameters are only used when they are defined by crystal symmetry. An approximate (isotropic) treatment of cell esds is used for estimating esds involving l.s. planes.
Refinement. Refinement of F^2^ against ALL reflections. The weighted R-factor wR and goodness of fit S are based on F^2^, conventional R-factors R are based on F, with F set to zero for negative F^2^. The threshold expression of F^2^ > 2sigma(F^2^) is used only for calculating R-factors(gt) etc. and is not relevant to the choice of reflections for refinement. R-factors based on F^2^ are statistically about twice as large as those based on F, and R- factors based on ALL data will be even larger.

### Fractional atomic coordinates and isotropic or equivalent isotropic displacement parameters (Å^2^)

**Table:**

	*x*	*y*	*z*	*U*_iso_*/*U*_eq_	
O1	−0.0416 (3)	0.95164 (11)	1.23851 (7)	0.0557 (5)	
O2	−0.0242 (3)	1.00698 (11)	0.89550 (9)	0.0653 (6)	
H2	−0.1156	1.0337	0.9049	0.098*	
O3	0.0626 (3)	0.71841 (10)	0.80452 (8)	0.0564 (5)	
H3A	0.0723	0.6798	0.7792	0.085*	
O4	0.2230 (3)	0.83520 (14)	0.67050 (8)	0.0713 (6)	
O5	−0.1210 (3)	0.82035 (12)	0.62841 (8)	0.0603 (5)	
O6	0.0212 (3)	0.72295 (11)	0.57042 (8)	0.0611 (5)	
O7	0.0666 (3)	0.85527 (12)	0.34091 (8)	0.0658 (6)	
H7	0.0474	0.8889	0.3117	0.099*	
O8	−0.1085 (3)	0.93713 (12)	0.40118 (10)	0.0690 (6)	
O9	0.0883 (3)	0.57919 (13)	0.73352 (10)	0.0763 (6)	
H2W	0.0463	0.5672	0.6951	0.114*	
H1W	0.2019	0.5690	0.7392	0.114*	
C1	−0.1098 (3)	0.96647 (15)	1.06144 (10)	0.0436 (5)	
H1A	−0.1831	0.9991	1.0313	0.052*	
H1B	−0.1630	0.9129	1.0624	0.052*	
C2	−0.1196 (4)	1.00276 (15)	1.13148 (11)	0.0520 (6)	
H2B	−0.0803	1.0585	1.1299	0.062*	
H2C	−0.2459	1.0015	1.1472	0.062*	
C3	−0.0006 (4)	0.95744 (14)	1.17876 (10)	0.0444 (5)	
C4	0.1676 (4)	0.92345 (15)	1.15279 (11)	0.0448 (5)	
H4A	0.2477	0.8984	1.1825	0.054*	
C5	0.2140 (3)	0.92622 (13)	1.08788 (10)	0.0374 (5)	
C6	0.4020 (3)	0.90065 (16)	1.06572 (11)	0.0477 (6)	
H6A	0.4626	0.8727	1.1022	0.057*	
H6B	0.4742	0.9481	1.0552	0.057*	
C7	0.3970 (3)	0.84573 (15)	1.00483 (10)	0.0458 (5)	
H7A	0.3440	0.7943	1.0174	0.055*	
H7B	0.5217	0.8361	0.9895	0.055*	
C8	0.2841 (3)	0.88213 (12)	0.94764 (10)	0.0339 (4)	
H8A	0.3439	0.9315	0.9320	0.041*	
C9	0.2692 (3)	0.82262 (13)	0.88998 (10)	0.0369 (4)	
H9A	0.2057	0.7750	0.9074	0.044*	
C10	0.4473 (4)	0.79267 (17)	0.85697 (12)	0.0535 (6)	
H10A	0.5027	0.7497	0.8829	0.064*	
H10B	0.5355	0.8361	0.8522	0.064*	
C11	0.3832 (4)	0.76156 (16)	0.78761 (12)	0.0549 (7)	
H11A	0.4528	0.7874	0.7522	0.066*	
H11B	0.4011	0.7038	0.7844	0.066*	
C12	0.1772 (3)	0.78255 (13)	0.78154 (10)	0.0418 (5)	
C13	0.1533 (3)	0.85407 (12)	0.83136 (9)	0.0356 (5)	
C14	0.2382 (4)	0.93041 (13)	0.80015 (11)	0.0478 (6)	
H14A	0.2240	0.9748	0.8304	0.072*	
H14B	0.1768	0.9424	0.7589	0.072*	
H14C	0.3666	0.9215	0.7917	0.072*	
C15	−0.0418 (3)	0.86831 (17)	0.85699 (10)	0.0447 (5)	
H15A	−0.0955	0.8170	0.8694	0.054*	
H15B	−0.1155	0.8907	0.8211	0.054*	
C16	−0.0500 (3)	0.92549 (15)	0.91754 (11)	0.0445 (5)	
H16A	−0.1738	0.9216	0.9367	0.053*	
C17	0.0888 (3)	0.90217 (12)	0.97298 (9)	0.0327 (4)	
H17A	0.0421	0.8512	0.9908	0.039*	
C18	0.0874 (3)	0.96102 (12)	1.03428 (9)	0.0343 (4)	
C19	0.1631 (4)	1.04641 (13)	1.01880 (11)	0.0494 (6)	
H19A	0.1587	1.0787	1.0586	0.074*	
H19B	0.0893	1.0710	0.9846	0.074*	
H19C	0.2881	1.0423	1.0036	0.074*	
C20	0.1216 (4)	0.80295 (14)	0.70948 (10)	0.0484 (6)	
C21	−0.0732 (4)	0.78122 (19)	0.68959 (11)	0.0589 (7)	
H21A	−0.0833	0.7233	0.6841	0.071*	
H21B	−0.1578	0.7976	0.7245	0.071*	
C22	−0.0565 (3)	0.78637 (15)	0.57212 (11)	0.0449 (5)	
C23	−0.1019 (4)	0.83901 (14)	0.51351 (10)	0.0453 (5)	
H23A	−0.0684	0.8941	0.5241	0.054*	
H23B	−0.2337	0.8375	0.5060	0.054*	
C24	−0.0045 (4)	0.81402 (14)	0.45007 (11)	0.0491 (6)	
H24A	0.1253	0.8064	0.4595	0.059*	
H24B	−0.0540	0.7629	0.4349	0.059*	
C25	−0.0252 (4)	0.87517 (15)	0.39558 (11)	0.0466 (5)	

### Atomic displacement parameters (Å^2^)

**Table:**

	*U*^11^	*U*^22^	*U*^33^	*U*^12^	*U*^13^	*U*^23^
O1	0.0697 (12)	0.0668 (11)	0.0306 (8)	−0.0100 (10)	0.0046 (8)	0.0002 (7)
O2	0.0894 (15)	0.0594 (11)	0.0469 (10)	0.0358 (11)	−0.0174 (10)	0.0003 (8)
O3	0.0850 (14)	0.0468 (9)	0.0373 (8)	−0.0218 (9)	0.0110 (9)	−0.0018 (7)
O4	0.0798 (14)	0.0982 (15)	0.0358 (9)	−0.0302 (13)	0.0077 (9)	0.0125 (9)
O5	0.0673 (12)	0.0787 (12)	0.0349 (8)	0.0154 (11)	0.0019 (8)	−0.0053 (8)
O6	0.0848 (14)	0.0542 (10)	0.0443 (9)	0.0136 (10)	0.0007 (9)	−0.0003 (8)
O7	0.0914 (15)	0.0696 (12)	0.0363 (8)	0.0176 (12)	0.0047 (10)	0.0010 (8)
O8	0.0811 (14)	0.0663 (12)	0.0597 (11)	0.0290 (12)	0.0072 (11)	0.0108 (9)
O9	0.0737 (14)	0.0851 (14)	0.0703 (12)	−0.0039 (13)	−0.0130 (12)	−0.0341 (11)
C1	0.0407 (12)	0.0587 (13)	0.0314 (10)	0.0081 (11)	0.0005 (9)	−0.0009 (9)
C2	0.0586 (15)	0.0618 (14)	0.0357 (11)	0.0119 (13)	0.0065 (11)	−0.0032 (10)
C3	0.0564 (14)	0.0447 (11)	0.0321 (10)	−0.0107 (11)	0.0000 (10)	−0.0025 (9)
C4	0.0513 (14)	0.0504 (12)	0.0328 (10)	−0.0016 (11)	−0.0084 (10)	0.0051 (9)
C5	0.0381 (11)	0.0396 (10)	0.0345 (10)	−0.0028 (9)	−0.0082 (9)	0.0011 (8)
C6	0.0396 (12)	0.0659 (14)	0.0378 (11)	0.0060 (11)	−0.0101 (10)	0.0008 (10)
C7	0.0392 (12)	0.0571 (13)	0.0411 (11)	0.0125 (11)	−0.0062 (10)	0.0020 (10)
C8	0.0311 (10)	0.0388 (10)	0.0319 (9)	0.0013 (9)	−0.0021 (8)	0.0020 (8)
C9	0.0383 (11)	0.0375 (10)	0.0349 (10)	0.0039 (9)	0.0009 (9)	0.0019 (8)
C10	0.0490 (14)	0.0601 (14)	0.0516 (13)	0.0175 (12)	0.0045 (11)	−0.0039 (11)
C11	0.0634 (17)	0.0563 (14)	0.0450 (13)	0.0122 (13)	0.0094 (13)	−0.0054 (11)
C12	0.0536 (14)	0.0412 (11)	0.0307 (10)	−0.0060 (10)	0.0060 (9)	0.0007 (9)
C13	0.0394 (11)	0.0387 (10)	0.0287 (9)	−0.0012 (9)	0.0012 (8)	0.0017 (8)
C14	0.0596 (15)	0.0401 (11)	0.0436 (12)	−0.0036 (12)	−0.0009 (11)	0.0081 (9)
C15	0.0370 (12)	0.0667 (14)	0.0303 (9)	0.0017 (11)	−0.0048 (9)	−0.0044 (10)
C16	0.0349 (11)	0.0643 (14)	0.0343 (10)	0.0118 (11)	−0.0054 (9)	−0.0039 (10)
C17	0.0320 (10)	0.0371 (10)	0.0289 (9)	−0.0002 (8)	−0.0023 (8)	0.0022 (7)
C18	0.0379 (11)	0.0362 (10)	0.0286 (9)	0.0023 (9)	−0.0016 (8)	0.0038 (8)
C19	0.0647 (16)	0.0405 (11)	0.0431 (12)	−0.0053 (12)	−0.0013 (11)	0.0029 (9)
C20	0.0634 (16)	0.0528 (13)	0.0290 (10)	−0.0045 (12)	0.0071 (11)	−0.0016 (9)
C21	0.0637 (17)	0.0807 (18)	0.0322 (11)	−0.0067 (15)	0.0042 (11)	−0.0002 (12)
C22	0.0436 (12)	0.0530 (13)	0.0380 (11)	−0.0041 (11)	−0.0010 (10)	−0.0058 (10)
C23	0.0470 (13)	0.0496 (12)	0.0394 (11)	0.0029 (11)	−0.0034 (10)	−0.0015 (9)
C24	0.0617 (15)	0.0470 (12)	0.0387 (11)	0.0075 (12)	−0.0039 (11)	−0.0013 (10)
C25	0.0501 (14)	0.0531 (13)	0.0366 (11)	0.0027 (12)	−0.0061 (10)	−0.0042 (10)

### Geometric parameters (Å, °)

**Table:**

O1—C3	1.236 (3)	C9—C10	1.536 (3)
O2—C16	1.436 (3)	C9—H9A	0.9800
O2—H2	0.8200	C10—C11	1.552 (4)
O3—C12	1.428 (3)	C10—H10A	0.9700
O3—H3A	0.8200	C10—H10B	0.9700
O4—C20	1.199 (3)	C11—C12	1.542 (4)
O5—C22	1.344 (3)	C11—H11A	0.9700
O5—C21	1.429 (3)	C11—H11B	0.9700
O6—C22	1.195 (3)	C12—C20	1.535 (3)
O7—C25	1.323 (3)	C12—C13	1.560 (3)
O7—H7	0.8200	C13—C15	1.526 (3)
O8—C25	1.199 (3)	C13—C14	1.542 (3)
O9—H2W	0.8502	C14—H14A	0.9600
O9—H1W	0.8500	C14—H14B	0.9600
C1—C2	1.527 (3)	C14—H14C	0.9600
C1—C18	1.536 (3)	C15—C16	1.540 (3)
C1—H1A	0.9700	C15—H15A	0.9700
C1—H1B	0.9700	C15—H15B	0.9700
C2—C3	1.486 (4)	C16—C17	1.548 (3)
C2—H2B	0.9700	C16—H16A	0.9800
C2—H2C	0.9700	C17—C18	1.568 (3)
C3—C4	1.443 (4)	C17—H17A	0.9800
C4—C5	1.343 (3)	C18—C19	1.552 (3)
C4—H4A	0.9300	C19—H19A	0.9600
C5—C6	1.498 (3)	C19—H19B	0.9600
C5—C18	1.526 (3)	C19—H19C	0.9600
C6—C7	1.522 (3)	C20—C21	1.514 (4)
C6—H6A	0.9700	C21—H21A	0.9700
C6—H6B	0.9700	C21—H21B	0.9700
C7—C8	1.532 (3)	C22—C23	1.499 (3)
C7—H7A	0.9700	C23—C24	1.511 (3)
C7—H7B	0.9700	C23—H23A	0.9700
C8—C9	1.523 (3)	C23—H23B	0.9700
C8—C17	1.543 (3)	C24—C25	1.498 (3)
C8—H8A	0.9800	C24—H24A	0.9700
C9—C13	1.536 (3)	C24—H24B	0.9700
			
C16—O2—H2	109.5	C15—C13—C14	112.4 (2)
C12—O3—H3A	109.5	C9—C13—C14	111.68 (19)
C22—O5—C21	116.3 (2)	C15—C13—C12	115.87 (19)
C25—O7—H7	109.5	C9—C13—C12	99.67 (16)
H2W—O9—H1W	115.0	C14—C13—C12	108.81 (16)
C2—C1—C18	113.05 (19)	C13—C14—H14A	109.5
C2—C1—H1A	109.0	C13—C14—H14B	109.5
C18—C1—H1A	109.0	H14A—C14—H14B	109.5
C2—C1—H1B	109.0	C13—C14—H14C	109.5
C18—C1—H1B	109.0	H14A—C14—H14C	109.5
H1A—C1—H1B	107.8	H14B—C14—H14C	109.5
C3—C2—C1	110.9 (2)	C13—C15—C16	113.31 (18)
C3—C2—H2B	109.5	C13—C15—H15A	108.9
C1—C2—H2B	109.5	C16—C15—H15A	108.9
C3—C2—H2C	109.5	C13—C15—H15B	108.9
C1—C2—H2C	109.5	C16—C15—H15B	108.9
H2B—C2—H2C	108.0	H15A—C15—H15B	107.7
O1—C3—C4	121.4 (2)	O2—C16—C15	109.54 (19)
O1—C3—C2	121.0 (2)	O2—C16—C17	111.77 (19)
C4—C3—C2	117.49 (19)	C15—C16—C17	112.59 (18)
C5—C4—C3	123.2 (2)	O2—C16—H16A	107.6
C5—C4—H4A	118.4	C15—C16—H16A	107.6
C3—C4—H4A	118.4	C17—C16—H16A	107.6
C4—C5—C6	120.4 (2)	C8—C17—C16	114.68 (16)
C4—C5—C18	122.8 (2)	C8—C17—C18	113.40 (17)
C6—C5—C18	116.67 (18)	C16—C17—C18	113.60 (17)
C5—C6—C7	112.63 (19)	C8—C17—H17A	104.6
C5—C6—H6A	109.1	C16—C17—H17A	104.6
C7—C6—H6A	109.1	C18—C17—H17A	104.6
C5—C6—H6B	109.1	C5—C18—C1	109.64 (16)
C7—C6—H6B	109.1	C5—C18—C19	105.83 (18)
H6A—C6—H6B	107.8	C1—C18—C19	110.34 (19)
C6—C7—C8	111.99 (18)	C5—C18—C17	108.04 (16)
C6—C7—H7A	109.2	C1—C18—C17	108.62 (17)
C8—C7—H7A	109.2	C19—C18—C17	114.26 (16)
C6—C7—H7B	109.2	C18—C19—H19A	109.5
C8—C7—H7B	109.2	C18—C19—H19B	109.5
H7A—C7—H7B	107.9	H19A—C19—H19B	109.5
C9—C8—C7	110.37 (17)	C18—C19—H19C	109.5
C9—C8—C17	108.86 (17)	H19A—C19—H19C	109.5
C7—C8—C17	109.39 (16)	H19B—C19—H19C	109.5
C9—C8—H8A	109.4	O4—C20—C21	120.7 (2)
C7—C8—H8A	109.4	O4—C20—C12	123.2 (3)
C17—C8—H8A	109.4	C21—C20—C12	116.1 (2)
C8—C9—C13	113.40 (17)	O5—C21—C20	110.1 (2)
C8—C9—C10	118.41 (19)	O5—C21—H21A	109.6
C13—C9—C10	104.11 (17)	C20—C21—H21A	109.6
C8—C9—H9A	106.7	O5—C21—H21B	109.6
C13—C9—H9A	106.7	C20—C21—H21B	109.6
C10—C9—H9A	106.7	H21A—C21—H21B	108.2
C9—C10—C11	103.9 (2)	O6—C22—O5	123.9 (2)
C9—C10—H10A	111.0	O6—C22—C23	126.5 (2)
C11—C10—H10A	111.0	O5—C22—C23	109.5 (2)
C9—C10—H10B	111.0	C22—C23—C24	113.2 (2)
C11—C10—H10B	111.0	C22—C23—H23A	108.9
H10A—C10—H10B	109.0	C24—C23—H23A	108.9
C12—C11—C10	106.6 (2)	C22—C23—H23B	108.9
C12—C11—H11A	110.4	C24—C23—H23B	108.9
C10—C11—H11A	110.4	H23A—C23—H23B	107.8
C12—C11—H11B	110.4	C25—C24—C23	112.2 (2)
C10—C11—H11B	110.4	C25—C24—H24A	109.2
H11A—C11—H11B	108.6	C23—C24—H24A	109.2
O3—C12—C20	108.3 (2)	C25—C24—H24B	109.2
O3—C12—C11	111.9 (2)	C23—C24—H24B	109.2
C20—C12—C11	112.3 (2)	H24A—C24—H24B	107.9
O3—C12—C13	107.28 (17)	O8—C25—O7	123.1 (2)
C20—C12—C13	113.75 (18)	O8—C25—C24	124.4 (2)
C11—C12—C13	103.30 (19)	O7—C25—C24	112.4 (2)
C15—C13—C9	107.79 (16)		
			
C18—C1—C2—C3	−55.5 (3)	C13—C15—C16—C17	−49.1 (3)
C1—C2—C3—O1	−148.7 (2)	C9—C8—C17—C16	−49.2 (2)
C1—C2—C3—C4	33.7 (3)	C7—C8—C17—C16	−169.87 (18)
O1—C3—C4—C5	177.8 (2)	C9—C8—C17—C18	178.04 (15)
C2—C3—C4—C5	−4.7 (4)	C7—C8—C17—C18	57.4 (2)
C3—C4—C5—C6	171.1 (2)	O2—C16—C17—C8	−78.2 (2)
C3—C4—C5—C18	−4.0 (4)	C15—C16—C17—C8	45.6 (3)
C4—C5—C6—C7	134.0 (2)	O2—C16—C17—C18	54.4 (2)
C18—C5—C6—C7	−50.7 (3)	C15—C16—C17—C18	178.24 (19)
C5—C6—C7—C8	52.3 (3)	C4—C5—C18—C1	−17.2 (3)
C6—C7—C8—C9	−175.34 (19)	C6—C5—C18—C1	167.61 (19)
C6—C7—C8—C17	−55.6 (3)	C4—C5—C18—C19	101.8 (2)
C7—C8—C9—C13	178.47 (18)	C6—C5—C18—C19	−73.4 (2)
C17—C8—C9—C13	58.4 (2)	C4—C5—C18—C17	−135.4 (2)
C7—C8—C9—C10	−59.1 (3)	C6—C5—C18—C17	49.4 (2)
C17—C8—C9—C10	−179.18 (19)	C2—C1—C18—C5	46.3 (3)
C8—C9—C10—C11	−159.65 (19)	C2—C1—C18—C19	−69.9 (2)
C13—C9—C10—C11	−32.7 (2)	C2—C1—C18—C17	164.16 (18)
C9—C10—C11—C12	5.4 (3)	C8—C17—C18—C5	−52.8 (2)
C10—C11—C12—O3	−91.9 (2)	C16—C17—C18—C5	173.95 (18)
C10—C11—C12—C20	146.2 (2)	C8—C17—C18—C1	−171.64 (17)
C10—C11—C12—C13	23.2 (2)	C16—C17—C18—C1	55.1 (2)
C8—C9—C13—C15	−62.0 (2)	C8—C17—C18—C19	64.7 (2)
C10—C9—C13—C15	168.0 (2)	C16—C17—C18—C19	−68.6 (2)
C8—C9—C13—C14	61.9 (2)	O3—C12—C20—O4	−156.6 (3)
C10—C9—C13—C14	−68.2 (2)	C11—C12—C20—O4	−32.6 (3)
C8—C9—C13—C12	176.70 (18)	C13—C12—C20—O4	84.3 (3)
C10—C9—C13—C12	46.7 (2)	O3—C12—C20—C21	24.4 (3)
O3—C12—C13—C15	−39.4 (3)	C11—C12—C20—C21	148.4 (2)
C20—C12—C13—C15	80.3 (2)	C13—C12—C20—C21	−94.7 (3)
C11—C12—C13—C15	−157.70 (19)	C22—O5—C21—C20	79.2 (3)
O3—C12—C13—C9	75.9 (2)	O4—C20—C21—O5	−13.1 (4)
C20—C12—C13—C9	−164.4 (2)	C12—C20—C21—O5	165.9 (2)
C11—C12—C13—C9	−42.4 (2)	C21—O5—C22—O6	7.3 (4)
O3—C12—C13—C14	−167.12 (19)	C21—O5—C22—C23	−174.2 (2)
C20—C12—C13—C14	−47.4 (3)	O6—C22—C23—C24	−12.4 (4)
C11—C12—C13—C14	74.6 (2)	O5—C22—C23—C24	169.1 (2)
C9—C13—C15—C16	55.8 (3)	C22—C23—C24—C25	−170.0 (2)
C14—C13—C15—C16	−67.6 (2)	C23—C24—C25—O8	0.3 (4)
C12—C13—C15—C16	166.44 (18)	C23—C24—C25—O7	177.2 (2)
C13—C15—C16—O2	75.9 (2)		

### Hydrogen-bond geometry (Å, °)

**Table:**

*D*—H···*A*	*D*—H	H···*A*	*D*···*A*	*D*—H···*A*
O2—H2···O8^i^	0.82	2.06	2.828 (3)	155
O3—H3A···O9	0.82	1.91	2.720 (2)	170
O7—H7···O1^ii^	0.82	1.91	2.716 (2)	167
O9—H2W···O2^iii^	0.85	2.08	2.884 (3)	158
O9—H1W···O1^iv^	0.85	1.95	2.795 (3)	174

Symmetry codes: (i) −*x*−1/2, −*y*+2, *z*+1/2; (ii) *x*, *y*, *z*−1; (iii) −*x*, *y*−1/2, −*z*+3/2; (iv) *x*+1/2, −*y*+3/2, −*z*+2.
